# Sialic acids in cancer biology and immunity—recent advancements

**DOI:** 10.1016/j.jbc.2025.110641

**Published:** 2025-08-28

**Authors:** Sandra J. van Vliet, Yvette van Kooyk

**Affiliations:** 1 Amsterdam UMC location Vrije Universiteit Amsterdam, Department of Molecular Cell Biology and Immunology, Amsterdam, the Netherlands; 2 Cancer Center Amsterdam, Cancer Biology and Immunology, Amsterdam, the Netherlands; 3 Amsterdam Institute for Immunology and Infectious Diseases, Cancer Immunology, Amsterdam, the Netherlands

**Keywords:** sialic acids, tumor microenvironment, antitumor immunity, antitumor therapy

## Abstract

Tumors frequently display an aberrant glycosylation profile, often characterized by a hyperexpression of sialylated glycan moieties. Here, we provide a holistic view on how sialylation alters cellular behavior in the tumor niche that not only includes tumor cells but also comprises the intricate interplay of cancer cells with stromal cells, such as fibroblasts and immune cells. We discuss how sialylation alters tumor cell biology, migration, and metastasis, as well as how it impacts local infiltration of immune cell subsets, their differentiation, and function *via* Siglec receptors. Recent advances in biomarker discovery and therapeutics to interfere in these processes through sialic acid inhibitors, mimetics, and targeted therapeutics will furthermore be highlighted.

An aberrant glycophenotype is a common feature of cancer cells and includes *de novo* expression of truncated *O*-glycans, as well as an overexpression of tetra-antennary *N*-glycans, fucosylated, and sialylated structures (1). Sialic acids are generally found at the nonreducing termini of glycoproteins and glycolipids, thus capping and shielding the underlying carbohydrate structure. Sialic acids represent a large family of >80 naturally occurring structures, derived from the negatively charged nine-carbon neuraminic acid backbone (2). The three main members of the sialic acid family are *N*-acetylneuraminic acid, *N*-glycolylneuraminic acid, and ketodeoxynononic acid. Humans only express the *N*-acetylneuraminic acid because of a mutation in the *cytidine monophospho-N-acetylneuraminic acid hydroxylase* (*CMAH*) gene and are thus unable to synthesize *N*-glycolylneuraminic acid, the analog found in most mammals (3). Interestingly, humans can obtain *N*-glycolylneuraminic acid from food and incorporate it in host tissues, leading to low-grade inflammation and tumor development (4, 5). While ketodeoxynononic acid is the major sialic acid variety in certain lower invertebrates (fish) and bacteria, it is only found in trace amounts in mammals. Interestingly, *N*-acetylneuraminic acid can be further modified through *O*-acetylation, *O*-methylation, and *O*-sulfation, leading to an even larger diversity of structures present in the human glycocalyx.

One of the most studied glycoproteins in cancer are the mucins (MUCs), proteins containing hundreds of glycosylation sites in their backbone. In normal tissue, MUCs are expressed in the apical membrane of polarized epithelial cells; however, cancer cells often lose their polarization, resulting in the presence of MUCs in the tumor microenvironment and the bloodstream (6). Moreover, these cancer-associated MUCs present a multitude of truncated O-glycans, including Tn and T antigens, as well as their sialylated forms, including sialyl-Tn, sialyl-T, and di-sialyl-T (an overview of glycan structures mentioned in the review is given in Fig. 1). In fact, several MUCs are currently used as biomarkers in the clinic, such as MUC1 (CA15-3) in breast cancer and MUC16 (CA125) in ovarian cancer.

**Figure 1:**
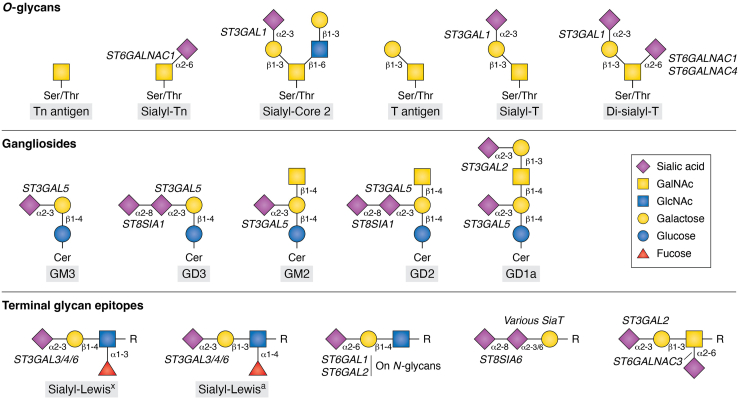
**Overview of common tumor-associated glycan structures.** Depicted are sialyltransferases and tumor-associated glycan structures, discussed in this review. Both common names, graphical representation (108), and appropriate sialyltransferases involved in the synthesis are included in the figure.

In this review, we will discuss how sialic acids affect cancer cell biology and antitumor immunity, focusing on developments within the oncology field of the last 5 years (Fig. 2). Sialic acids are currently connected to many classical hallmarks of cancer, including the induction of cancer stemness, replicative immortality, and resistance to apoptosis. These aspects will be more elaborately discussed in the paragraphs later. Moreover, the modulatory role of sialic acids to interact with immune receptors such as selectins and Siglecs and their migratory and immune modulatory functions will be highlighted.

**Figure 2:**
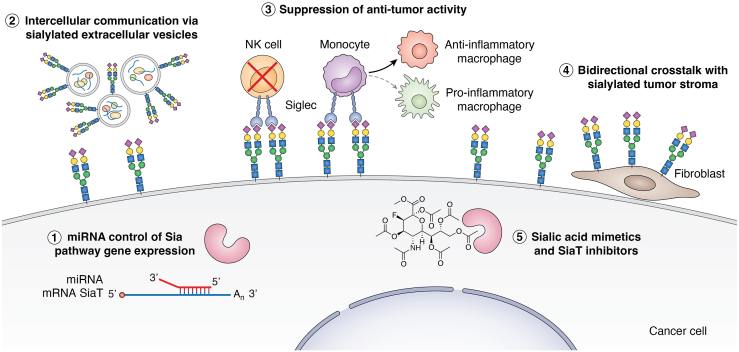
**Recent developments in the sialic acid axis in cancer cell biology and immunity.** Research over the last years has highlighted several new aspects of sialic acid–based modulation of the tumor microenvironment, including (1) the miRNA-mediated control of expression of sialylation (Sia)-related genes, (2) the communication *via* sialylated EVs, (3) the active suppression of antitumor immunity by tumor-derived sialic acids, (4) the bidirectional influence and effects of stromal cell sialylation in modulating the cancer and immune cell activation, and (5) the development of novel sialyltransferase (SiaT) inhibitors and mimetics to counteract the detrimental effects exerted by tumor-derived sialic acids. The sialylated *N*-glycan is shown as an example, and other sialylated glycans may be involved in the mechanisms depicted above. EV, extracellular vesicle.

## Regulation of sialic acid expression at the tumor site

Many tumor types exhibit abnormal expression of sialylated glycoproteins and lipids, with alterations in both the levels as well as the type of structures found on the cancer cells. This aberrant expression is even more prominent in necrotic core regions of especially breast, thyroid, and liver tumors, reflected by a conserved pattern of sialylated biantennary *N*-glycans that lack core fucose (7). Hypersialylation of cancer cells is generally attributed to a dysregulation in the expression of multiple sialyltransferases, often linked to the differentiation status of the tumor cells or the activation of oncogenic signaling pathways (8, 9). Next to alterations in sialyltransferase expression levels, also the availability of the sugar donor CMP–sialic acid is a crucial regulatory hub in maintaining a high sialylation profile in tumors. Thus, metabolic rewiring in cancer cells provides another mechanism governing sialic acid levels. Low levels of phosphoglycerate dehydrogenase in breast ductal carcinoma are associated with metastasis and tumor aggressiveness. Interestingly, loss of phosphoglycerate dehydrogenase activates the hexosamine pathway and increases *de novo* sialic acid biosynthesis. As a result, the integrin αvβ3 is hypersialylated, facilitating the disseminative phenotype (10). Alcohol and its oncogenic metabolite acetaldehyde were recently shown to dysregulate the proper localization of glycosyltransferases to the Golgi, thus leading to abnormally glycosylated proteins (11).

Lately, miRNAs have been implicated in regulating the sialic acid phenotype of tumors. miRNAs are small noncoding RNAs involved in post-transcriptional control of gene expression. They are mostly known for silencing gene expression by destabilizing mRNA and for mediating mRNA destruction. Indeed, miR-21, miR-30e, and miR-26b all negatively correlate with downregulated ST6GALNAC1 expression in colorectal cancer (12). Nevertheless, several miRNAs, such as miR-221-5p or miR-212-5p, actually amplify ST6GAL1 expression, a process dependent on the miRNA-binding proteins AGO2 and FXR1 (13). In contrast, the miRNAs that target ST6GAL2 levels are mostly downregulatory. The enzyme CMP *N*-acetylneuraminic acid synthetase (CMAS) that activates and generates the CMP–sialic acid for conjugation is also under miRNA control. miRNA regulation of CMAS is bidirectional, yet in pancreatic cancer, the upregulatory miRNAs prevail, which, in conjunction with ST6GAL1 expression, is responsible for the α2-6 sialic acid high phenotype in pancreatic cancer cells (14). Similarly, upregulatory miRNAs targeting CD98hc (part of the amino acid transporter LAT-1), ST3GAL1, and ST3GAL2 are enriched in melanoma, forming a coregulatory network of the carrier protein CD98hc and the ST3GAL1/2 enzymes responsible for its sialylation (15).

During tumor development, many different cell types accumulate in the tissue and create a niche in which tumor cells grow along with stromal cells and various immune cells. Cancer stem cells are a subpopulation of cancer cells, resembling actual stem cells. These cells display a unique ability to self-renew and have an enhanced ability to grow in an anchorage-independent manner, rendering them more tumorigenic and metastatic. These cancer stem cells appear to be characterized by a distinct glycosylation profile (elegantly reviewed in Ref. (16)). For example, breast cancer stem cells selectively express α2-3 sialylated core2 *O*-linked glycans, whereas sialyl-Lewis antigens and FUT-3 are enriched in a non-stem cell and differentiation population, both in breast and colon cancer cells (17, 18). The chromosomal region harboring the stemness transcription factor Sox2 shows frequent gains in copy number, among others, in ovarian cancer (19). This same region also contains the Sox2 target ST6GAL1, suggesting that coamplification of Sox2 and ST6GAL1 further intensifies ST6GAL1 expression in the tumor. In general, cancer stem cells are thought to have a higher plasticity, allowing them to switch across different cell states, contributing to therapy resistance and tumor progression. Interestingly, especially sialylated glycosphingolipid species (gangliosides) have been implicated in controlling cancer cell plasticity. For instance, polysialylation of gangliosides by ST8SIA6 supports anchorage-independent cell growth (20).

Not only tumor cell sialylation but also sialylation of stromal cells attributes to the growing tumor niche (21, 22). Interestingly, the interaction between cancer cells, stromal fibroblasts, and the immune system appears to have reciprocal effects, with both tumor and stromal cell sialylation influencing immune cell activation (discussed later), but also *vice versa*, the attraction of immune cells to the tumor site may actively alter the glycan profile of the cancer cells. The cytokine interleukin 13 (IL-13), produced by M2-like macrophages, is able to promote STAT6 phosphorylation, thereby inducing ST6GALNAC1 and sialyl-Tn expression (23). IL-1β and IL-6 promote the expression of ST6GAL1 *via* an NF-κB and STAT-3-mediated pathway, respectively (24). Thus, local inflammation may reprogram the cellular glycosylation profile *via* NF-κB–STAT signaling and epigenetic modifications of glycosyltransferase genes (25).

In general, recent research has highlighted the multilayered complexity in maintaining the sialylation phenotype of tumors, ranging from regulation of sialylation pathway gene expression and availability of CMP–sialic acid donors to the intricate interplay between cancer cells and surrounding stromal and immune cells.

## Sialic acids in endorsing the malignant tumor phenotype

Changes in glycosylation patterns have been associated with malignant transformation and clinical outcomes in several cancer types, prompting ongoing research into the mechanisms involved and potential clinical applications. Recently, transcriptomic analysis of glycosylation-related genes and pathways, using publicly available bulk and single-cell transcriptomic datasets from tumor samples and cancer cell lines, identified genes and pathways strongly associated with different tumor types, which may represent novel diagnostic biomarkers (26). For example, the expression of the gene set reflecting the ‘‘Synthesis of CMP-Sialic acid,’’ which leads to the generation of the glycan donor used by sialyltransferases, is associated with better survival in colorectal and liver cancer but with lower survival in sarcoma, uveal melanoma, and lung, pancreatic, and breast cancer. Remarkably, this seems to be related to the expression in normal tissue, as sialylation levels are already very high in healthy liver and colon compared with normal melanocytes, lung, pancreas, and breast tissue. We previously reported that the knockout of CMAS, a key enzyme in this pathway, leads to a more aggressive tumor in a mouse model of colorectal cancer (27).

α2-6 sialylation and especially ST6GAL1 have a strong tumor-promoting role in among others, pancreatic cancer (28, 29) and glioblastoma (30). In the well-established KC (K-ras^LSL.G12D/+;^ Pdx-1-Cre) mouse model of pancreatic cancer, a pancreas-specific deletion of *ST6Gal1* delayed tumor formation and accompanying fibrosis, indicating a strong tumor-promoting role for this enzyme and for *N*-linked α2–6 sialic acids (29). Mechanistically, this has been linked to the modification of receptors on the cell surface that regulate cell growth and apoptosis.

The TNF–TNFR1 axis plays a crucial role in balancing cancer cell death and survival. While cell-surface TNFR1 initiates a survival program upon TNF binding, TNFR1 internalization triggers caspase-mediated apoptosis. ST6GAL1-mediated α2–6 sialylation of TNFR1 promotes cancer cell survival by preventing TNFR1 oligomerization, high-affinity TNF binding, and subsequent internalization (28). This process was fully dependent on α2–6 sialylation and was not occurring upon α2–3 sialylation of TNFR1 (31). Interestingly, FAS and PDGF receptor β may be undergoing a similar type of regulation through ST6GAL1 and α2–6 sialic acids (30, 31). Next to being a cancer cell survival factor, ST6GAL1 also protects tumors from hypoxia through the modulation of cancer cell metabolism (32). Interestingly, ST6GAL1 improves both oxidative and glycolytic pathways in ovarian cancer cells under hypoxia, thus sustaining the overall fitness of ovarian cancer even under stressed conditions.

Not only α2–6 sialylation of Gal appears to stimulate cancer malignancy but also α2–6 sialylated GalNAc has a tumor-promoting role. In lung cancer cells, ST6GALNAC3 controls the mRNA stability and protein expression of the transferrin receptor protein 1 and is a critical regulator of tumor cell iron metabolism (33). Sialyl-Tn expression on MUC16 enhances the aggressive phenotype of pancreatic ductal adenocarcinoma (PDAC) tumor cells, increasing epidermal growth factor receptor (EGFR) interactions and triggering AKT and glycogen synthase kinase-3β oncogenic signaling (34).

Interestingly, also opposite effects have been reported. Blocking the sialidase NEU1, thus increasing cellular sialylation, hampered proliferation and apoptosis in the breast cancer cell lines MCF-7 and MDA-MB-231 (35).

Next to controlling tumor cell growth and apoptosis, sialic acids are also involved in regulating tumor angiogenesis. ST3GAL1-mediated sialylation of vasorin enhances its binding to transforming growth factor beta 1 (TGF-β1), thereby promoting TGF-β1 signaling and expression of angiogenic genes (36). ST3GAL1 is also a transcriptional target of TGF-β1, suggesting a self-sustaining feedback loop promoting tumor vascularization.

In ovarian cancer, expression of ST8SIA1 and ganglioside synthesis are particularly found in mesenchymal cancer cells and associated with worse prognosis (37). In contrast, globosides are mainly detected in epithelial cells, suggesting that glycosphingolipids control tumor plasticity and epithelial-to-mesenchymal transition. In intrahepatic cholangiocarcinoma, the stem cell–like subset expressed higher levels of the ganglioside GD2 (38). In cell line models, high expression of GD2 was linked to a higher sphere-forming ability, as well as drug resistance. Interestingly, in intrahepatic cholangiocarcinoma patients, high gene expression of GD3S, the glycosyltransferase involved in synthesizing GD2, was associated with lymph node metastasis, indicating that GD2 may also be relevant in patients.

## Sialic acids controlling tumor cell invasion and metastasis

Sialic acids are well known to contribute to invasion and metastasis by promoting cell detachment, enhancing, and ultimately driving the spread of malignant cells. Classical rolling and adhesion receptors, like the selectins and their sialyl-Lewis antigen ligands, are important mediators of cancer cell motility and metastasis. Expression of sialyl-Lewis^X^ and sialyl-Lewis^A^ is dependent on the presence of several sialyltransferases, most notably ST3GAL3 and ST3GAL4, and on the β1,6-*N*-acetylglucosaminyltransferase GCNT3, at least in gastrointestinal cancers (39, 40, 41). The tumor aggressiveness of prostate cancer cell lines has been linked to the expression of GALNT5 and ST3GAL6, mediating the synthesis of α2–3 sialic acids and especially sialyl-Lewis^A^ on MUCs in prostate cancer cell lines (42, 43). In prostate cancer patients, elevated levels of sialyl-Lewis^A^ were particularly prominent in stage IV tumors (43), providing more evidence for sialyl-Lewis antigens and their role in tumor metastasis. Recently, de-*O*-acetylation of sialic acids was shown to be an integral requirement for selectin binding and subsequent cell migration (44).

Interestingly, some tumors also express receptors for sialic acids. Especially, Siglec-15 is overexpressed by several tumor types, including hepatocellular, colorectal, thyroid, and cervical cancer, and promotes the cancer cell’s migratory capacity (45, 46, 47). Several mechanisms have been put forward explaining the role of Siglec-15 in tumor progression. In cervical cancer cell lines, Siglec-15 protects the cells from apoptosis *via* a mitogen-activated protein kinase/extracellular signal–regulated kinase–dependent pathway involving mitochondrial retrograde signaling (45). In hepatoma cells, Siglec-15 acts *via* its association with the known tumor (stem cell) biomarker CD44. The CD44–Siglec-15 interaction is mediated by α2–6 sialic acids on the CD44 molecule, which improves the stability of CD44 and prevents its lysosomal degradation (46). Whether this involves rare CD44 cancer-specific splice forms and expression of sialyl-Tn remains to be determined (48). Siglec-15 also engages α2–6/α2–8 sialic acids on the EGFR in thyroid cancer cells (47). This interaction likewise stabilizes the EGFR protein and establishes the migratory phenotype.

EGFR itself is a sialylation target of the ST6GAL1 enzyme, regulating the mechanosignaling and ligand-dependent activity of EGFR. ST6GAL1 is upregulated in many tumor types, including prostate cancer, where it facilitates bone metastasis, a process that could be counteracted using sialyltransferase inhibitors (49). α2–6 sialylation of EGFR *N*-glycans increases its retention at the cell surface and inhibits its lysosomal degradation, which culminates in EGFR–integrin crosstalk facilitating the migratory and invasive phenotype (50, 51). Mechanistically, integrin mechanical forces, tension, and adhesion were coordinated by an ERK-mediated signaling pathway, while spreading and invasion seemed to depend on PI3K–AKT (50). EGFR signaling can also be indirectly regulated *via* the interaction with ST3GAL1-modified *O*-glycosylation of neuropilin (52). Silencing of ST3GAL1 diminished EGF–EGFR binding and signaling, resulting in reduced migration and invasion of breast cancer cells.

Also the truncated *O*-glycan sialyl-Tn and its synthesizing enzyme ST6GALNAC1 promote migration and metastasis (53, 54), a process linked to hypoxia (55). In a *Kras* and *Trp53* mutant lung cancer tumor model, knockout of ST6GALNAC1 reduces sialyl-Tn on MUC5AC and results in less liver metastasis *in vivo* (53). Interestingly, some sialylated glycans actually seem to have an antitumor effect and protect from tumor dissemination. For instance, sialyl-T on MUC1 suppresses ovarian cancer cell seeding at the peritoneal site (56). Also ST8SIA1 levels are negatively correlated to muscle invasiveness and the grading of bladder cancer tumors (57). ST8SIA1 overexpression in bladder cancer cell lines resulted in the inhibition of JAK2 and STAT3 phosphorylation and decreased expression of Bcl2, cyclin D1, and the metalloprotease MMP2. Consistently, loss of certain sialylated epitopes likewise accelerated tumor progression. In human high-grade serous ovarian cancer, elevated expression of the sialidase NEU4 is associated with tumor metastasis and poor survival, which in this tumor type might be linked to specific desialylation of N^196^ in EGFR (58). A similar trait is found in bladder cancer, where NEU3 activates ERK and PI3K pathways leading to invasion and a heightened malignant phenotype (59). As both NEU3 and NEU4 have an affinity preference for cleaving α2–3 and α2–8 sialic acids over α2–6 sialylated structures, this suggests the existence of a dichotomy between sialyl-Lewis and α2–6 sialylated epitopes (generated by ST6GAL1 and ST6GALNAC1) promoting invasiveness and, in contrast, certain other α2–3 moieties, like sialyl-T, protecting against tumor spread to distal organs. This apparent dichotomy warrants further, in-depth exploration.

## Sialic acid and intercellular communication *via* extracellular vesicles

Extracellular vesicles (EVs) are cell-derived membranous vesicles that facilitate cellular communication by transferring messengers, DNA/RNA, and proteins (60). Glycomics analysis and cell-based glycoengineering have revealed that EVs are heavily glycosylated and surrounded by a dense glycocalyx (61, 62). Yet, the EV glycosylation patterns can differ markedly depending on the cancer cells they originate from (62, 63). In general, EV glycan profiles are linked to their biodistribution and uptake by different recipient endothelial, epithelial, or immune cell subsets (64).

EVs from several cancer types, including prostate, ovarian, and bladder cancer, have high levels of both α2–3 and α2–6 sialic acids (65, 66, 67, 68). EV-associated sialoglycans have been recognized for their ability to boost tumor cell migration and metastasis by enabling a premetastatic niche formation. These processes especially involve specific sialylated epitopes on integrins. Introducing Sialyl-Tn expression on a non–small cell lung cancer cell line activates a TP53 and tumor suppressor–activated pathway 6–mediated pathway that enhances EV production (68). These EVs selectively encase FAK, which upon transfer enhanced the motility of the recipient cells. EVs from carrying sialyl-Lewis^X^-decorated integrin a3 specifically target E-selectin on endothelial cells, disrupting tight junction barriers by inducing the disintegration of occluding tight junctions (69). Overall, this results in increased vascular permeability, which facilitates bladder cancer cell extravasation and metastasis. In addition, sialylated bladder cancer EVs also prime the lung and liver tissue for incoming cancer cells, a process that could be attributed to sialylated β1 integrin in the vesicles and its binding to fibronectin in the extracellular matrix (65). Mechanistically, sialylated EVs reprogram surrounding normal epithelial cells, thereby establishing a seeding niche for incoming metastatic bladder cancer cells.

## Sialic acid and the induction of an immunosuppressive tumor microenvironment

During oncogenesis, tumor cells acquire mutations that result in the expression of neoantigens, which can be sensed by the immune system to build up an antitumor immune response. Release of these antigens by tumor cells allows the antigens to be captured by dendritic cells (DCs), which in the presence of immunogenic signals can trigger T-cell responses in the tumor-draining lymph node (70). The enhanced sialylation machinery of tumor cells alters the glycosylation of these antigens, inducing antigen-specific tolerogenic programming in DCs and leading to activation of regulatory T cells and dampening of effector T-cell responses (70, 71). Sialoglycan-covered antigens are mainly recognized by Siglec receptors on DCs that signal through immune inhibitory cytoplasmic tails and induce antitumor programming of DCs by changing the DC cytokine secretion profile and by altering the expression of maturation markers. Similarly, removal of sialic acids from tumor cell–based vaccines has been shown to enhance antitumor immune responses by increasing DC-mediated T-cell activation (72). Thus, eliminating sialic acids from antigens or the tumor microenvironment may represent a potential strategy to augment the immunogenicity of cancer-associated antigens and improve antitumor immunity.

Hypersialylation in both tumor and stroma is not only involved in hampering DC-induced T cell-mediated killing, but it has also been shown to drive the differentiation of tumor-infiltrating monocytes to monocyte-derived macrophages, tumor-associated macrophages (TAMs), and myeloid-derived suppressor cells (73, 74). In PDAC, upregulation of ST3GAL1 and ST3GAL4 in tumor cells was identified as the main contributor to the synthesis of Siglec-7 and Siglec-9 ligands. These Siglec ligands could crosslink Siglec-7/-9 on monocytes and drive these monocytes to TAM differentiation accompanied by secretion of suppressive cytokines, such as IL-10, thereby creating a full immunosuppressive environment. Therefore, the sialic acid–Siglec axis has been defined as a glycoimmune checkpoint in which the immune inhibitory cytoplasmic tail of these Siglec receptors behaves similarly to the PD-1 immune checkpoint to reduce antitumor immunity (75, 76). Selective expression of ST3GAL4 in PDAC stromal cells drives this high sialylation phenotype and also has the potency to drive Siglec-7/-9-mediated monocyte to TAM differentiation, thus paralyzing myeloid cells within the stromal compartment (21). ST3GAL4 has also been identified to be associated with sialyl-Lewis^X^ expression on endothelial cells, creating a binding site for L-selectin on T cells and mediating extravasation of T cells to home to secondary lymphoid organs or the tumor site. Alternatively, expression of sialyl-Lewis^X^ on T cells serves as a ligand for P-selectin on endothelial cells, allowing T-cell migration.

Sialylation is also able to reduce NK cell–mediated cytotoxicity of tumor cells (77), a process enhanced by sialic acid deacetylation (78). T cells hardly express Siglec transcripts; instead, they acquire Siglec-7 and -9 from interacting myeloid cells in the tumor microenvironment *via* trogocytosis, which impairs their activation and effector function when present in the tumor (79, 80). Also tumor-infiltrating Siglec-9-positive CD8 T cells are functionally inhibited upon interaction with sialoglycans (81). Siglec-10 and -9 are similarly increased on memory T cells compared with naïve T cells, and mouse studies on the Siglec-10 homolog, Siglec-G, identified that the interaction with CD24 sialoglycans functions as a coinhibitory axis that limits CD8 T-cell function by metabolic rewiring (82). However, also Siglec ligands on T cells themselves have been shown to regulate their activation status (83). Overall, these findings demonstrate an overarching suppression of sialic acids by interacting with Siglecs on TAMs to dampen T-cell and NK-cell responses.

Although these cellular interactions consist of *trans-*interactions between Siglecs and sialic acids, *cis*-interactions also play a role in dampening immune cell function (84). Currently, it is not known whether the sialoglycan ligands and carrier proteins on tumor and stromal cells that engage Siglec-7 and -9 are similar. Often MUCs have been identified to carry these ligands; however, in melanoma also, CD24, CD43, and the gangliosides GD2, GD3, and GM3 are sialylated (85). CD24 has been identified as a Siglec-10 ligand in melanoma and is associated with poor prognoses and enhanced tumor growth and metastasis *in vivo* (86). Mechanistically, the CD24–Siglec-10 interaction acts as an innate checkpoint, hampering the phagocytosis of cancer cells by TAMs (87).

These findings illustrate that sialylation of tumor and stromal cells may locally alter the immune cell infiltration, differentiation, and activity in the tumor niche, all to increase tumor growth and disease severity. Interestingly, immune cells in turn can affect cancer cell sialylation (23), suggesting a self-sustaining feedback loop to promote immune evasion by the tumor.

## Sialic acid targeting and resistance to therapy

An elevated tumor sialylation profile also has strong implications for therapy, and research over the past few years has highlighted that sialylation and especially ST6GAL1 convey resistance to several types of cancer therapy, including immunotherapy, small-molecule inhibitors, radiotherapy, and chemotherapy. ST6GAL1 is upregulated upon chemotherapy, preventing cancer cell apoptosis and thus reducing treatment efficacy (88). Acquired resistance to the androgen receptor antagonist enzalutamide occurs in nearly all patients with metastatic prostate cancer. This resistance is accompanied by an upregulation of ST6GAL1 and α2–6 sialylation and could be reversed using enzalutamide in combination with a sialic acid inhibitor (89). Along that line, removal of sialic acid O-acetylation sensitizes colon and lung cancer cells to EGFR inhibition (90) and may involve sialic acid modifications of ABC drug transporters on the cell surface (91).

Resistance may also occur through the concomitant upregulation of sialic acid receptors, such as Siglec-9. In esophageal squamous cell carcinoma, radiotherapy induces Siglec-9 expression and polarization of M2-like macrophages. These macrophages subsequently further augment the radioresistance and prime the immunosuppressive tumor microenvironment (92).

Nevertheless, the highly sialylated tumor microenvironment also offers opportunities for therapy. The multicellular strategies by which sialic acids promote immune evasion make them very interesting to modulate for future immunotherapy. Various studies have shown the beneficial effects of interfering in the sialic acid–Siglec axis, leading to reduced tumor growth and increased survival benefit. Preclinical models have highlighted that reduction of sialic acids by interfering with the expression of sialyltransferases or transporters increases percentages of effector CD8 T cells and reduces regulatory T cells. Tumor-targeted sialidases coupled to a HER2 antibody enhanced antitumor immunity in murine breast and melanoma models (93, 94). Even in combination with immune checkpoints like anti-PD-1 and anti-CTLA-4, targeted sialidases show enhanced beneficial effects compared to treatment with the sialidase alone. Phase I–II clinical trials have started in PDAC with the bisialidase (E-602), which effectively desiaylates the tumor microenvironment including T cells and NK cells, and increases effector CD8 T cells at the tumor site. Also, the combination with anti-PD-(L)1 in different cancer types is currently being explored (clinical trial NCT05259696). Targeting stromal cell sialylation could improve antitumor immunity even further and revert its suppression by increasing T-cell influx at the tumor site (21, 22). Combining tumor desialylation with anti-PD-1 showed reduction of tumor growth, illustrating that integrating sialic acid removal and current immune checkpoints may have added effect in improving survival benefit (95, 96).

Single-cell RNA sequencing and spatial transcriptomics of patients with glioblastoma multiforme treated with neoadjuvant anti-PD-1 therapy identified unique monocyte-derived tumor-associated macrophage subpopulations with functional plasticity that highly expressed the immunosuppressive *SIGLEC9* gene and preferentially accumulated in the nonresponders to anti-PD-1 treatment (97). Mechanistically, targeting the murine counterpart Siglec-E in a mouse model activated both CD4^+^ T cells and CD8^+^ T cells through antigen presentation, secreted chemokines, and costimulatory factor interactions (93). Also here, Siglec-E deletion synergized with anti-PD-1/PD-L1 treatment to improve antitumor efficacy, again indicating that Siglec-9 is an immune-checkpoint molecule on macrophages that can be targeted to repolarize macrophages to enhance anti-PD-1/PD-L1 therapeutic efficacy of difficult-to-treat cancers, like glioblastoma multiforme and PDAC. Alternatively, anti-Siglec antibodies could act as therapeutics to interfere in tumor growth by dampening interactions with sialic acids (98). Vice versa, sialic acid inhibitors or mimetics have been designed to inhibit sialyltransferases or target high-affinity Siglec receptors, respectively (99, 100). These sialic acid inhibitors were able to strongly block tumor growth in a melanoma model when applied locally, which resulted in increased CD8 T-cell activation and infiltration and killing of the tumor (101, 102). Currently, also, combination therapies using sialic acid blockade and chemoimmunotherapy have provided novel opportunities for antitumor therapy (103).

Next to targeted sialidases or developing high-affinity Siglec ligands that direct receptors to the lysosome for degradation (79), sialic acid–targeted cyclodextrin-based nanoparticles can be used to reprogram tumor-associated macrophages into inflammatory macrophages by delivering CSF/1R siRNA in the nanoparticles (104). Other therapeutic strategies include the use of chimeric antigen receptor T cells (CAR-T cells) to target the sialylated gangliosides GM2 or GD2. Anti-GD2 and GM2 CAR-T cells expressing IL-7 and CCL19 for GM2-positive solid cancer in patients with neuroblastoma revealed that long-term outcomes are achieved with GD2-directed CAR-T cell therapy (105, 106).

## Conclusions and future directions

Sialylation of the tumor microenvironment has been shown to have an enormous impact not only on tumor invasion and metastasis but also on locally imprinting immunity by engaging Siglecs as immune inhibitory receptors. A lot of attention has been given to ST6GAL1 and α2–6 sialylation of *N*-glycans, whereas other sialyltransferases and sialylated glycans have been far less studied. New developments in mass spectrometry glycomics have revolutionized the determination of glycans and their carrier proteins in the tumor context, implying an enormous potential for the field in terms of large-scale glycoproteomics profiling (107). Moreover, with current novel technologies present, such as single-cell RNA-Seq and spatial analysis, new discoveries may reveal different sialylation signatures as predictors for immune responder or nonresponder status. This will add information to the current exploration of the diversity of immune landscape analysis between patients, their immune stimulatory or inhibitory identity, and the spatial accumulation of immune cells in the tumor niche.

The further identification of sialoglycans, including their carrier proteins, as ligands for specific Siglecs could benefit biomarker discovery to select patients who are most likely to benefit from therapy. Preclinical experiments have already demonstrated that interfering in the Siglec–sialic acid axis may benefit response rates of future therapies by combining these with existing immune checkpoint therapies to maximize therapeutic benefit. It is, however, important to fully understand the biological effects of desialylation, as its impact is affecting a wide variety of immune cells and may vary according to tumor type.

Whereas the biology of certain sialic acids is well understood, the individual contribution of both *cis-* and *trans*-interactions with Siglecs in the tumor microenvironment is largely unexplored. We should also not forget that certain sialic acids or sialyltransferases may have a protective function. Due to the multitude of cellular functions that develop in time between tumor, stroma, and immune cells, the impact may not be unidirectional but likely bidirectional. Overall, these are important research directions for future exploration.

## Conflict of interest

The authors declare that they have no conflicts of interest with the contents of this article.
